# Multimodal Computational Modeling of Visual Object Recognition Deficits but Intact Repetition Priming in Schizophrenia

**DOI:** 10.3389/fpsyt.2020.547189

**Published:** 2020-11-23

**Authors:** Pejman Sehatpour, Anahita Bassir Nia, Devin Adair, Zhishun Wang, Heloise M. DeBaun, Gail Silipo, Antigona Martinez, Daniel C. Javitt

**Affiliations:** ^1^College of Physicians and Surgeons, New York State Psychiatric Institute, Columbia University, New York, NY, United States; ^2^Nathan Kline Institute for Psychiatric Research, Orangeburg, NY, United States; ^3^School of Medicine, Yale University, New Haven, CT, United States; ^4^Department of Biomedical Engineering, The City College of New York, City University of New York, New York City, NY, United States

**Keywords:** closure, connectivity, ERP, fMRI, perception, priming, vision

## Abstract

The term perceptual closure refers to the neural processes responsible for “filling-in” missing information in the visual image under highly adverse viewing conditions such as fog or camouflage. Here we used a closure task that required the participants to identify barely recognizable fragmented line-drawings of common objects. Patients with schizophrenia have been shown to perform poorly on this task. Following priming, controls and importantly patients can complete the line-drawings at greater levels of fragmentation behaviorally, suggesting an improvement in their ability to perform the task. Closure phenomena have been shown to involve a distributed network of cortical regions, notably the lateral occipital complex (LOC) of the ventral visual stream, dorsal visual stream (DS), hippocampal formation (HIPP) and the prefrontal cortex (PFC). We have previously demonstrated the failure of closure processes in schizophrenia and shown that the dysregulation in the sensory information transmitted to the prefrontal cortex plays a critical role in this failure. Here, using a multimodal imaging approach in patients, combining event related electrophysiological recordings (ERP) and functional magnetic resonance imaging (fMRI), we characterize the spatiotemporal dynamics of priming in perceptual closure. Using directed functional connectivity measures we demonstrate that priming modifies the network-level interactions between the nodes of closure processing in a manner that is functionally advantageous to patients resulting in the mitigation of their deficit in perceptual closure.

## Introduction

Schizophrenia is associated with sensory functions that are impaired, but also with functions that are paradoxically preserved. The pattern of impairment/preservation does not depend upon brain region, but rather on function within brain region. A key example of this dissociation is within the ventral stream visual system where some functions, such as perceptual closure (1–3) are impaired (4–7), but other functions, such as illusory contour processing (8), are paradoxically intact (9). We have suggested that the key difference between these two processes is that illusory contour processing occurs early following stimulus presentation (~170 ms) as detected using event-related potentials (ERP) and depends only upon the “feedforward sweep” of information from retina to ventral stream visual cortex (lateral occipital cortex, LOC), which is mediated primarily by the parvocellular visual system (8).

In contrast, perceptual closure occurs later (~270 ms) and depends upon not only feedforward input to LOC but also on network-level interaction that involves magnocellularly based activation of dorsal visual stream (DS) (10), followed by activation of both prefrontal (PFC) and hippocampal (HIPP) regions (6, 11–13). Because of generator geometry, activity in lateral inferior PFC and HIPP project poorly to the scalp and thus can be assessed better using fMRI or intracranial recordings than ERP. Patients show less dorsal stream activation than controls (14–17), leading to impaired activation of both PFC and HIPP (6).

The attenuation of feedback activation (18) through these regions accounts for the impairment in perceptual closure, as well as reduced ventral stream activity in schizophrenia as measured by parallel fMRI and ERP-based approaches (6). Furthermore, deficits in perceptual closure correlated significantly with cognitive function as reflected in the Perceptual Organization Index (POI) and Processing Speed Index (PSI) of the Wechsler Adult Intelligence Scale (WAIS-III) (19), along with clinical symptoms as reflected in scores on the Positive and Negative Syndrome Scale (PANSS) (20), indicating their importance to global function in schizophrenia (6).

At the time when we performed our initial studies of perceptual closure, an additional, paradoxical finding was that whereas closure thresholds were reduced overall, patients nevertheless showed relatively intact ability to take advantage of both literal and repetition priming in perceptual closure (1, 5). In literal priming, subjects are given a word that may or may not correspond to the object being shown. If it does correspond, then performance is improved as reflected in the ability to detect objects at a more fragmented level. In repetition priming, a stimulus is repeated, which also permits it to be identified subsequently at a more fragmented level. Both processes are considered to reflect interaction between HIPP and LOC, such that the priming procedures activate visual templates in HIPP that are then used by LOC to facilitate object identification.

Indeed, beta coherence between HIPP and LOC can be detected using intracranial electrodes (13) to closable vs. non-closable objects, supporting the role of top-down information transfer. Despite worse performance overall, as reflected in the need for less fragmented images, patients nevertheless showed the same degree of shift in closure level following priming as did controls, suggesting relatively intact HIPP-LOC interaction. Because of this interaction, object recognition following priming occurs as part of the initial feedforward sweep of information to LOC, which occurs at the time interval of the visual N1 (~170 ms) (21), rather than depending upon recurrent projects from dorsal stream to PFC to LOC, which is indexed by the later Ncl component.

We have previously shown that impaired closure processing in patients results from a network failure consequent to the initial dorsal stream phase of processing (4, 6). Here, we used a multimodal imaging approach to evaluate neural mechanisms underlying priming and their relative function in Sz patients vs. controls. Subjects viewed both novel and repeated stimuli, permitting assessment of priming effects. In ERP studies, we investigated the relative amplitudes of the visual P1, which reflects initial dorsal stream activation (16, 22); the N1, which reflects initial processing within LOC (9, 23); and the Ncl, which reflects recurrent processing involving dorsal stream, PFC and LOC. In fMRI, we evaluated integrity of activation within dorsal stream, LOC, PFC and HIPP to unprimed and repeat stimuli across groups, and used Granger causality (24–26) to evaluate patterns of interaction between these regions.

Overall, we hypothesized that in patients, both P1 and Ncl amplitudes would be reduced to unprimed stimuli, reflecting impaired dorsal stream contributions to the perceptual closure process, and that these deficits would be mirrored in impaired dorsal stream, PFC, and LOC activation to unprimed stimuli in schizophrenia. However, we hypothesized that N1 modulation to primed vs. unprimed stimuli would be relatively intact, reflecting preserved interaction between HIPP and LOC, and that activation patterns to primed stimuli would therefore be relatively intact in LOC to primed vs. unprimed stimuli. In Grainger causality, we predicted a loss of normal interaction between dorsal stream and PFC and between PFC and LOC in schizophrenia, but with preserved function within LOC and LOC-HIPP interaction. Overall, in keeping with the theme of this issue, the manuscript addresses how dysfunction within early sensory pathways, such as the visual magnocellular/dorsal stream pathway, contributes to higher order cognitive dysfunction in schizophrenia.

## Methods

### Participants

Data were collected in two separate experiments. In experiment 1 (ERP), and experiment 2 (fMRI) the same 19 male patients meeting DSM-IV criteria for schizophrenia and 21 healthy volunteers of similar age participated. Experiment one consisted of a single ERP session and experiment two was part of a larger fMRI study.

Patients were recruited from inpatient and outpatient facilities associated with the Nathan Kline Institute for Psychiatric Research. Informed consent was obtained after full explanation of procedures. Diagnoses were obtained using the Structured Clinical Interview for DSM-IV (SCID) (27). Healthy volunteers with a history of SCID-defined Axis I psychiatric disorder were excluded. Subjects were excluded if they had any neurological or ophthalmologic disorders that might affect performance or met criteria for alcohol or substance dependence within the last 6 months or abuse within the last month.

Patient and control groups did not differ significantly in age (patients, 37.3 ± 11.5 years; controls, 39.3 ± 8.5 years). The Positive and Negative Syndrome Scale (PANSS) total score was 71.82 ± 13.4 (*n* = 16). All patients but one, were receiving antipsychotics with twelve patients receiving atypical antipsychotics, two patients receiving typical antipsychotics, and three patients receiving a combination of atypical and typical antipsychotics. Chlorpromazine equivalents were 1,026 ± 871.7 mg/day. Duration of illness was 16.85 ± 10.7 years.

### Stimuli and Task

Methods were as described previously for ERP and fMRI studies (6, 12, 21). Briefly, fragmented line drawings of natural and man-made objects were drawn from the normed Snodgrass and Vanderwart picture set (28, 29). From these images, segments containing black pixels were randomly and cumulatively deleted to produce seven incrementally fragmented versions of each picture (30). Level 1 refers to the complete picture and Level 7 to the most fragmented version, where the proportion of deleted segments for any level equals [1–0.7^(level−1)^]. A set of “scrambled pictures,” serving as control stimuli for the fMRI study (6, 12), was generated by dividing the images into 16 × 16 segments, which were then scrambled.

### Stimulus Presentation

For ERP, stimuli were presented on an Iiyama Vision Master Pro 502 monitor located 143 cm from the subject. Images subtended an average of 4.8° (±1.4°) of visual angle in the vertical plane and 4.4° (±1.2°) in the horizontal plane. For fMRI, stimuli were delivered through a mirror system mounted on the head coil that reflected a projection screen behind the scanner.

### Timing of Stimulus Presentation for the ERP Study

For ERP, images were presented in accordance with the ascending method of limits (AML), from least complete to most complete (21). Based on previous studies of closure (5) using the same stimuli levels, 6 through 3 were used here. Each image appeared for 750 ms, followed by a blank screen for 800 ms. Then a “Y|N” response prompt appeared for 200 ms, followed by a blank screen for 2,200 ms. Subjects were instructed to press one button when they recognized the image as an object and another when they did not. Following “No” responses, subjects were presented with the next most complete image of the same picture and were again cued for a response. Following “Yes” responses, the picture sequence was terminated and subjects were required to verbally name the picture. The experiment consisted of 20 blocks, each block containing 10 different picture sequences, of which 5 were presented only once and 5 were presented twice (i.e., 15 picture sequences per block). Repeated picture sequences consisted of the identical fragmented images as when initially presented. The positions of the to-be repeated picture sequences were randomly selected. The number of picture sequences intervening between initial and repeated presentations was either one or two, determined at random. Subjects were encouraged to take breaks between blocks whenever they deemed it necessary to maintain high concentration and prevent fatigue.

### Timing of Stimulus Presentation for the fMRI Study

For fMRI, each image appeared for 500 ms, followed by a blank screen for 500 ms resulting in a stimulus onset asynchrony of 1 sec. Using the AML procedure outlined for the ERP stimulus presentation, the modal level of closure for each participant was determined prior to scanning; these images served as the primed condition here. Each stimulus block consisted of stimulus runs (9 TRs) of primed stimuli at the level of visual closure, unprimed stimuli at the level of visual closure and scrambled stimuli. The scanning sessions performed for these stimulus conditions consisted of 195 TRs containing three stimulus blocks. Four TRs of rest were shown at the beginning of each stimulus block and in between stimulus runs. The order of stimulus runs within each of the stimulus blocks was initially randomized, and the resulting order was used for all participants.

### EEG Data Acquisition and Analysis

Continuous EEG was acquired using an ANT (Enschede, The Netherlands) system with 64 scalp electrodes, average referenced and digitized at 512 Hz. Data were analyzed using BESA version 5.3 (Brain Electric Source Analysis, MEGIS Software GmbH). Electrode channels were subjected to an artifact criterion of ±120 μV from −100 to 500 ms. The vertical and horizontal electro-oculograms (HEOG and VEOG) were, in addition, visually inspected for blinks and large eye movements. For each subject, epochs were calculated for a time window from 100 ms pre to 500 ms post-stimulus and baseline-corrected relative to the pre-stimulus period. Accepted trials were then averaged separately for each condition to compute the VEP. *A priori* analysis (4, 12, 21) tested between-group differences in amplitude of the ERP components P1, N1, and Ncl within predetermined spatial and temporal windows (6, 12) (see figure legends and statistical analyses section). Between-group analyses for ERP were performed using repeated-measures multivariate analysis of variance (rmMANOVA) for each identified ERP component (P1, N1, Ncl). All tests of statistical significance were two-tailed with preset alpha level of *p* < 0.05. Analyses were conducted using SPSS software (SPSS Inc, Chicago, Il).

### fMRI Data Acquisition and Analysis

All functional and structural scans were performed using a 3T Siemens TIM Trio magnetic resonance scanner at the Nathan Kline Institute. Functional scans contained 36 axial slices, with TR = 2,000 ms, TE = 30 ms, and voxel size = 2.5 × 2.5 × 2.8 mm, with a 0.7 mm gap. High-resolution structural scans were performed with a 3-D magnetization prepared rapid acquisition gradient echo (MPRAGE) sequence, having 192 sagittal slices with TR = 2,500 ms, TE = 3.5 ms, FA = 8°, and voxel size = 1 × 1 × 1 mm. Image pre-processing was performed using SPM8 (http://www.fil.ion.ucl.ac.uk/spm/), and run under MATLAB 2010A. Functional images were first corrected for timing differences between slices using a windowed Fourier interpolation to minimize their dependence on the reference slice. The images after slice timing correction were then motion-corrected and realigned to the first image within each run, or discarded if estimates for peak motion exceeded 3 mm in any directions of the three translations and/or 2 degrees in any directions of the three rotations. The corrected images were coregistered and normalized to a standard MNI template by warping each subject's SPGR image to the MNI template ICBM152 and then warping each functional image to the subject specific SPGR image and resampled at a resolution of 3 × 3 × 3 mm^3^ per voxel. Images were then spatially smoothed using a Gaussian-kernel filter with a full width at half maximum of 8 mm.

We analyzed the functional image data acquired during task performance using SPM8 by two levels: the individual level (the first-level) to detect task-related activity within each individual participant; the group level (the second-level) to detect random effect of task-related activity within and between diagnostic groups. For first-level analysis we used the general linear model (GLM) in SPM8 where linear model regressors were generated by convolving the canonical hemodynamic response function (HRF) with each of the box car functions derived from the onsets and durations of the presentations of each stimulus condition. The model was estimated using the Restricted Maximum Likelihood (ReML) algorithm and then task-related T contrast images were generated using SPM8 contrast manager. Based on the previous studies of closure (6, 12, 13), regions of interest (ROI) at dorsal visual stream, fusiform gyrus, prefrontal cortex and hippocampal formation were then used for the next level of analyses which looked at differences between conditions and groups.

#### Directed Functional Connectivity Analysis

We used the Granger Causality (24) Index (GCI) as implemented in BrainVoyager QX3 (Brain Innovations, Maastricht, The Netherlands) to assess directed network influences across a set of regions identified in the second-level analysis, where the task-related activities showed significant differences between the diagnostic groups.

Based on the prediction theory outlined by (31), Granger causality uses the principle of temporal precedence to identify the direction of causality using the information in the data (32). That is, given two time series *x*[n] and *y*[n], we can identify the influence of *x* on *y* and vice versa. A measure of linear dependence *F*_*x,y*_ between *x*[n] and *y*[n] implementing Granger causality in terms of vector autoregressive (VAR) models was introduced by (33). A discrete zero-mean vector time-series *x*[n] = (*x*_1_[n], …, *x*_M_[n])^T^ can be modeled as a VAR of order *p* as follows (34):

$$\begin{array}{l} x\left[n\right]=\;-\;\overset{p}{\underset{i=1}{\sum }}A\left[i\right]x\left[n-i\right]+u\left[n\right] \end{array}$$

where *u*[n] is (multivariate) white noise. The matrices *A*[i] are called the autoregression (AR) coefficients as they regress *x*[n] onto its own past. As described in (25, 35), the VAR model can be considered as a linear prediction model that predicts the current value of *x*[n] based on a linear combination of the most recent past *p*-values. As such, the current value of *x*_*i*_[n] is predicted by a linear combination of its own past and that of the other components. This shows the utility of VAR model within the context of Granger causality. Given two time-series *x*[n] and *y*[n], one can compute the linear dependence between series *x* and *y*, with linear directed value from *x* to *y* (*F*_*x*→*y*_) being > 0 if the past values of *x* improve the prediction of current values of *y*. Likewise, linear directed value from *y* to *x* (*F*_*y*→*x*_) would be > 0 if the past values of *y* improve the prediction of current values of *x*. According to (33), much of the linear dependence can be contained in the undirected instantaneous influence *F*_*x*.*y*_ which quantifies the improvement in the prediction of the current value of *y* given the current value of *x* (or vice versa) in a linear model already containing their past values. Here, we computed the Granger Causality Maps (GCM) for each given reference ROI by calculating the influence measures *F*_*x*→*y*_, *F*_*y*→*x*_ and *F*_*x*.*y*_ from the average time-course of the voxels in the ROI (as *x*) and the voxel time-course (as *y*) for every voxel. We then calculated the influence difference term (*F*_*x*→*y*_–*F*_*y*→*x*_) for every voxel to form the difference-GCM (dGCM), mapping the influence to and from the ROI over the brain, with positive values in the difference term (index) indicating influence from *x* to *y* and negative values indicating influence from *y* to *x*. The thresholds on the maps were computed using bootstrap and false discovery rate (25). Considering that GCIs might not be normally distributed, we used the Wilcoxon signed rank method to test whether the medians of the GCIs were significantly different from zero for each connection of the selected two regions, each diagnostic group; we used Wilcoxon rank sum method to test whether the medians of the GCIs were significantly different between two diagnostic groups on each connection (36). The data were represented by the median and the inter-quartile range (IQR).

### Clinical Variables

Several relevant neuropsychological measures were administered. These included the Perceptual Organization (POI), the Processing Speed (PSI) Indices from the WAIS-III (19); the Working Memory Index (WMI) from the WMS-III (37); and the Brief Visuospatial Memory Test (BVMT-R) (38) which assesses retention of visual memory over time. The Positive and Negative Syndrome Scale (PANSS) (20) was used for symptom assessment.

## Results

### Behavior

**Figure 1:**
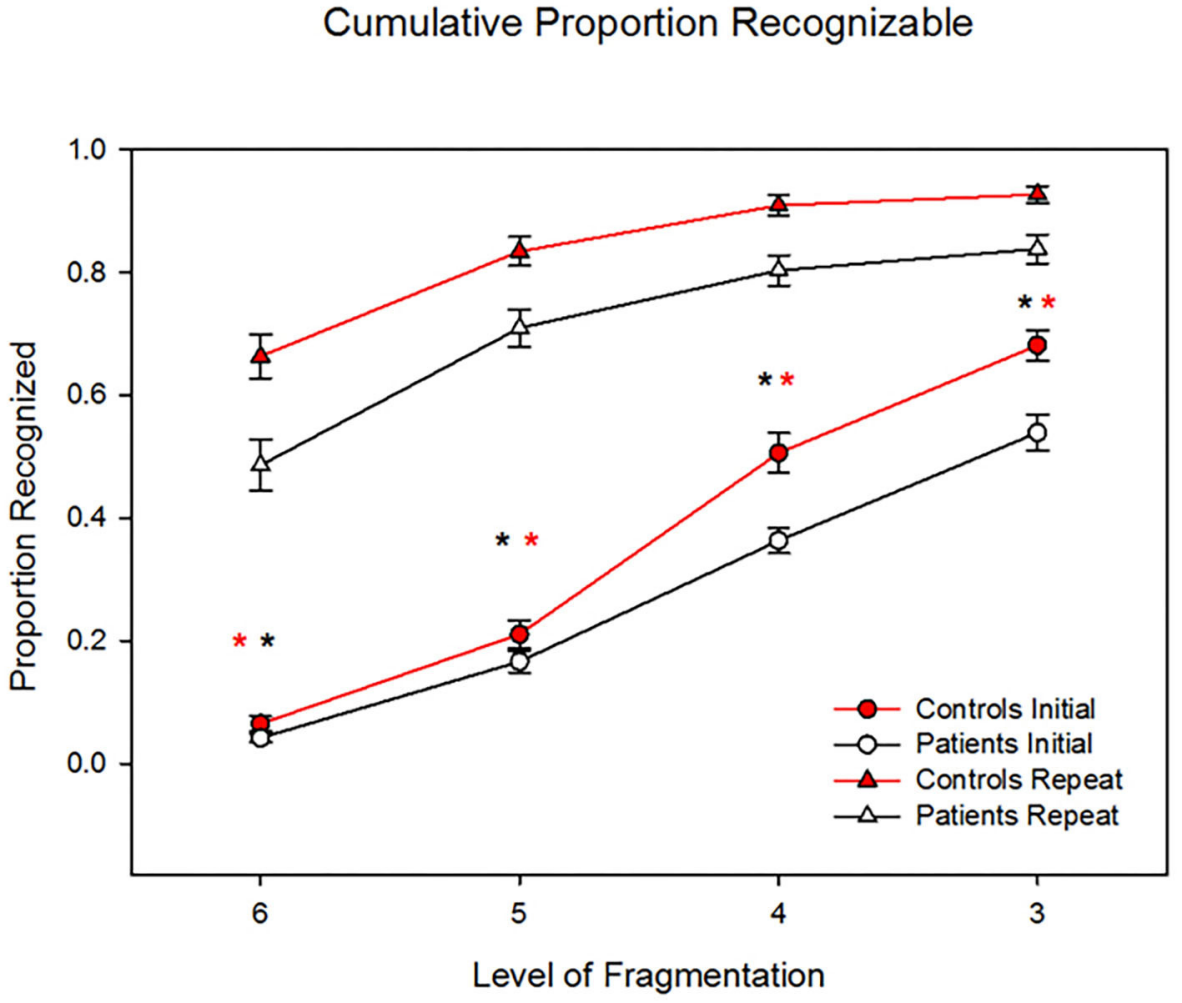
Behavioral results showing patients' significantly reduced identification vs. controls' to both novel (*P* = 0.005) and repeated (*P* = 0.009) stimuli. However, the two groups showed similar benefit to repetition priming across levels, such that the group X repetition effect was non-significant (*p* = 0.15). *p < 0.05.

Patients showed significantly reduced identification vs. controls to both novel (F_1,44_ = 8.77, *P* = 0.005) and repeated (F_1,44_= 7.47, *P* = 0.009) stimuli (Figure 1). Across all levels, patients performed significantly worse (F_1,44_ = 13.2, *p* = 0.001, d = 1.1). The level X group (F_3, 42_ = 0.15) and repeat X group (linear effect F_1,44_ = 2.42, *p* = 0.13, d = 0.47) were both non-significant. Analyses were potentially influenced by “floor” effects in patients at level 6 (initial), and “ceiling” effects in controls at level 3 (repeat). When analyses were repeated to focus on the two middle levels, the between-group difference remained significant (F_1,44_ = 11.6, *p* = 0.001, d = 1.02) and the repeat X group effect remained non-significant (F_1,44_ = 2.61, *p* = 0.11, d = 0.49).

### ERP

**Figure 2:**
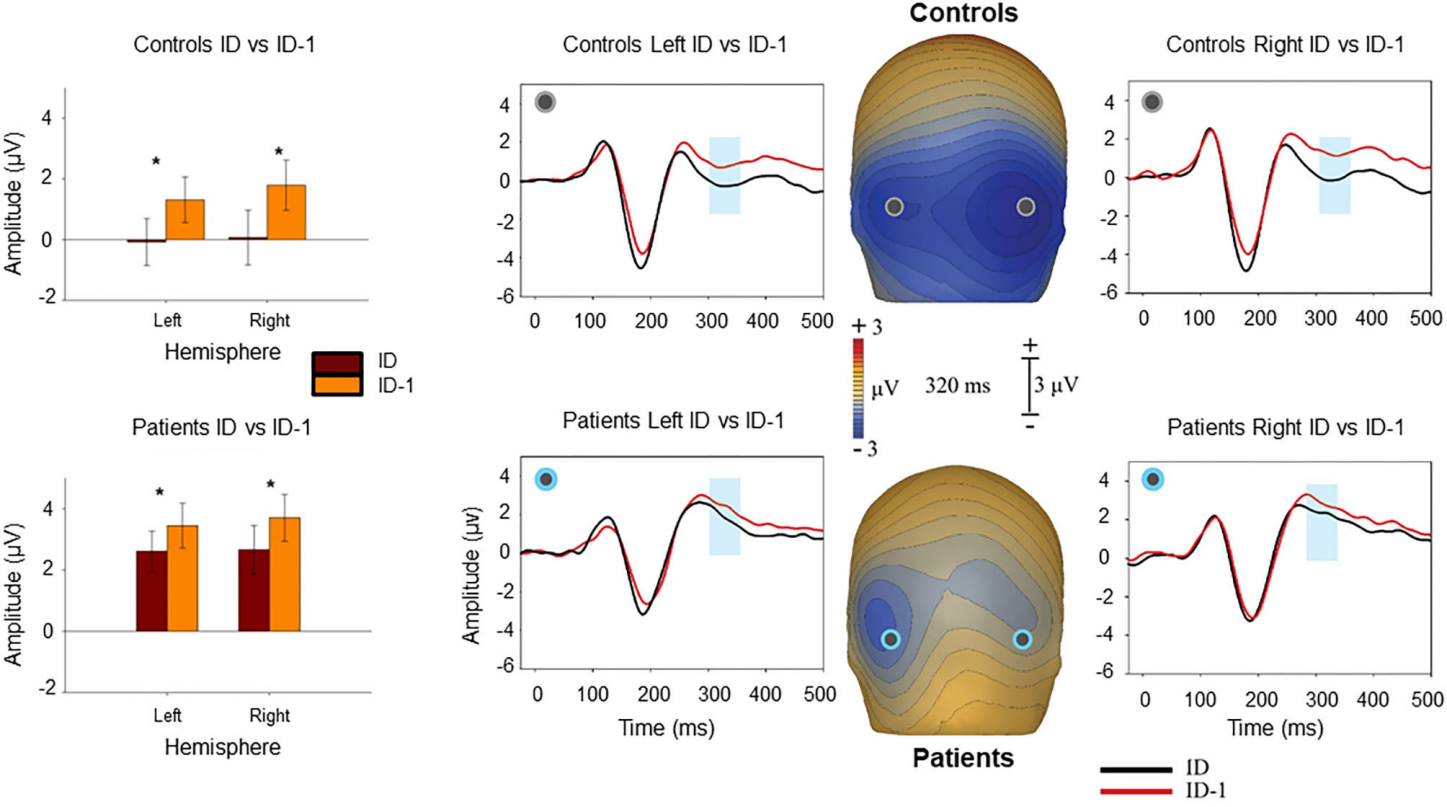
Voltage maps at 320 ms (peak Ncl activity) illustrate the relative negativity over lateral occipital scalp for novel image sequences at the level of identification (ID) vs. the prior image in the sequence (ID-1) stimuli. The graphs show scalp recording from two representative lateral occipital electrodes (PO7/PO8). The blue ribbon in the graphs show the tested window of time (300–340 ms) when the responses to the ID stimulus condition (in black) produced significantly larger negativity compared to the ID-1 (in red). The bar-charts show significant differences in the amplitude of responses to the ID and ID-1 in each hemisphere for controls and patients. *p < 0.05.

As reported previously (11, 12), closure was associated with increased negativity (“Ncl”) over visual object identification regions starting at ~250 ms and persisting to 500 ms, with maximal differential activity centered at 320 ms (Figure 2). Patients showed significantly reduced negativity over the interval relative to controls at the point of closure (F_1,37_ = 6.08, *p* = 0.018), but not the level prior (F_1,37_ = 1.93, *p* = 0.17). The between-group difference in Ncl amplitude was also significant to repeat stimuli (F_1,37_ = 4.48, *p* = 0.041).

**Figure 3:**
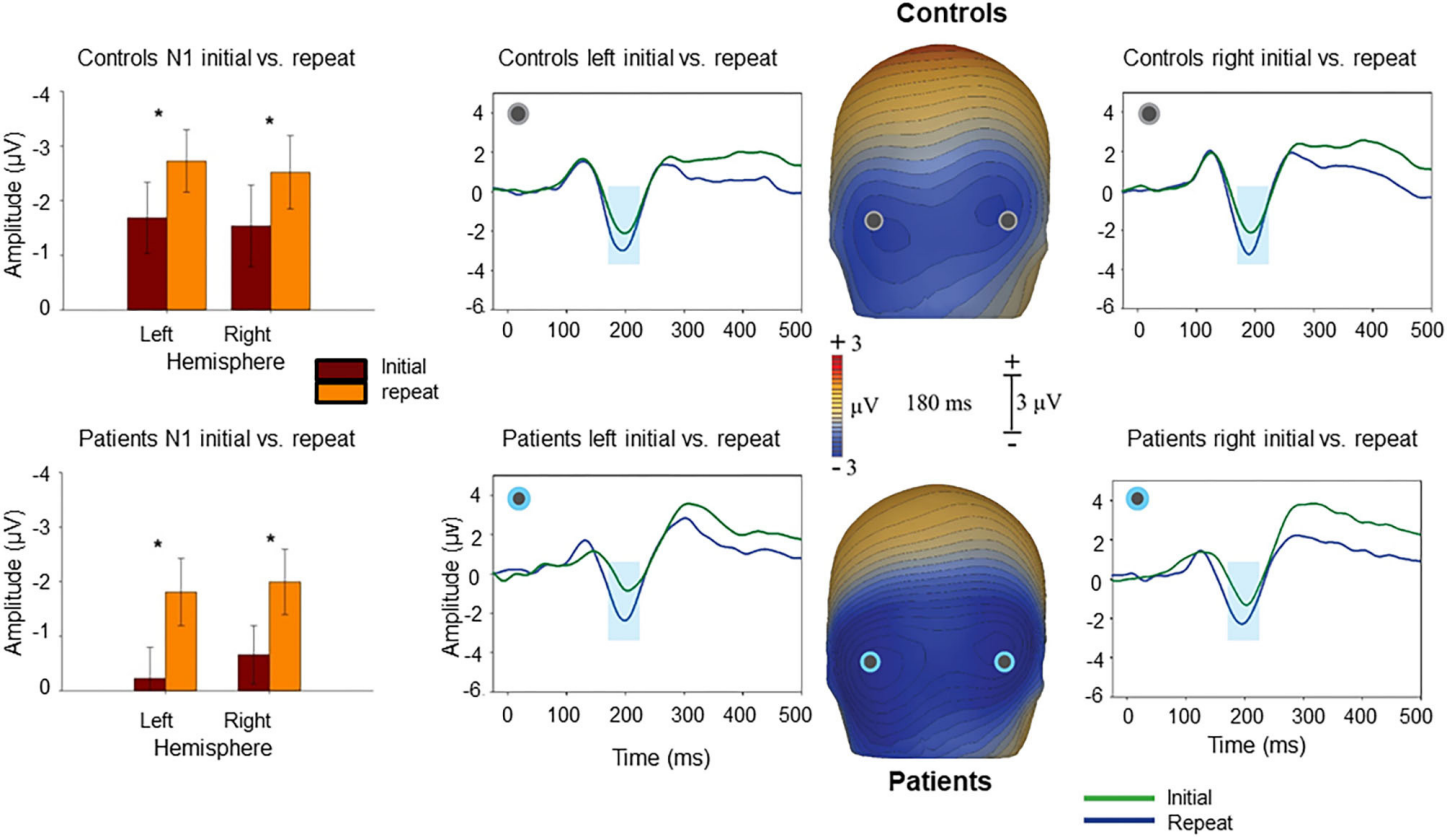
Voltage maps at 180 ms (peak N1 activity) illustrate the relative negativity over lateral occipital scalp for “Repeat” vs. the “Initial” presentations. The graphs show scalp recording from two representative lateral occipital electrodes (PO7/PO8). The blue ribbon in the graphs show the tested window of time (170–200 ms) when the responses to the “Repeat” stimulus condition (in blue) produced significantly larger negativity compared to the “Initial” (in green). The bar-charts show significant differences in the amplitude of responses to the “Repeat” and “Initial” in each hemisphere for controls and patients. *p < 0.05.

By contrast, repetition effects were primarily manifest within the latency range of N1 (170–200 ms) as reported previously (21) (Figure 3), such that larger N1 responses were observed at level of identification for “Repeat” images, than for images at that same level of fragmentation prior to priming “Initial” (F_1,37_ = 5.97, *p* = 0.019). The group X repeat interaction was non-significant (repeat X group F_1,37_ = 0.05, *p* = 0.8), suggesting similar repetition effects on N1 across groups. A group X effect type (N1 for repeated vs. Ncl for novel) showed a significant group X effect interaction (F_1,37_ = 18.1, *p* = 0.022).

**Figure 4:**
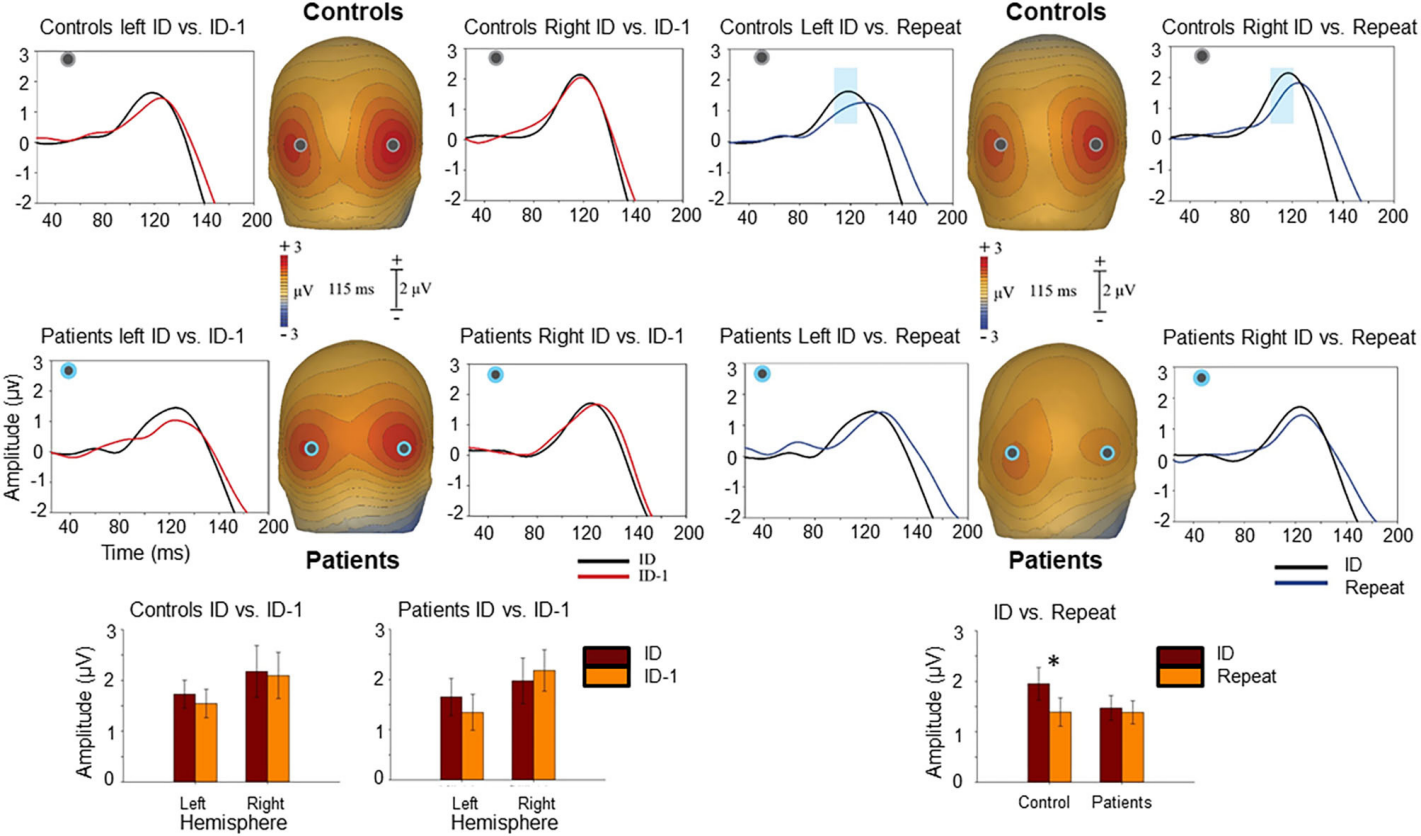
Voltage maps at 115 ms (peak P1 activity) illustrate the ERP responses to the ID and ID-1 images (left) as well as to the ID and “Repeat” images (right) over posterior scalp. The graphs show scalp recording from two representative occipital electrodes (PO5/PO6). The blue ribbon in the graphs show the tested window of time (100–120 ms) when the responses to the ID stimulus condition (in black) produced significantly larger positivity compared to the “Repeat” (in blue). This difference was only observed in controls. The bar-charts show significant differences in the amplitude of responses to the ID and ID-1 in each hemisphere for controls and patients (lower left) and for ID and “Repeat” across two hemispheres for controls and patients (lower right). *p < 0.05.

N1/Ncl reponses over ventral stream were preceded by P1 responses over dorsal stream. P1 responses were not significantly affected by level of fragmentation (*p* = 0.82). However, there was a significant effect of repetition with smaller P1 amplitudes to repeated stimuli in controls (F_1,20_ = 6.81, *p* = 0.017) but not in patients (F_1,17_ = 0.15, *p* = 0.7) (Figure 4). In controls, P1 amplitudes to initial stimuli correlated significantly with Ncl (*r* = 0.65, *p* = 0.001), whereas this relationship was lost in patients (*r* = 0.26, *p* = 0.29) (Table 1).

**Table 1:** Correlations between ERP measures P1, N1, and Ncl.

	**Closure Ncl**		**Repeat Ncl**		**Closure N1**		**Repeat N1**		**Closure P1**		**Repeat P1**	
**Patients**	***r***	***P*-value**	***r***	***P*-value**	***r***	***P*-value**	***r***	***P*-value**	***r***	***P*-value**	***r***	***P*-value**
Closure Ncl	–	–	0.86^**^	**0.001**	0.56^*^	**0.01**	0.78^**^	**0.001**	0.26	0.29	0.13	0.62
Repeat Ncl			–	–	0.42	0.08	0.73^**^	**0.001**	0.18	0.48	0.33	0.18
Closure N1					–	–	0.81^**^	**0.001**	0.26	0.29	0.09	0.73
Repeat N1							–	–	0.31	0.21	0.34	0.16
Closure P1									–	–	0.60^*^	**0.01**
Repeat P1											–	–
**Controls**
Closure Ncl	–	–	0.93^**^	**0.001**	0.60^**^	**0.001**	0.59^**^	**0.001**	0.65^**^	**0.001**	0.42	0.06
Repeat Ncl			–	–	0.49^*^	**0.02**	0.52^*^	**0.02**	0.57^*^	**0.01**	0.34	0.13
Closure N1					–	–	0.84^**^	**0.001**	0.48^*^	**0.03**	0.29	0.21
Repeat N1									0.40	0.07	0.17	0.47
Closure P1									–	–	0.84^**^	**0.001**
Repeat P1											–	–

* *p < 0.05*;

** *p < 0.01*.

*Bold values highlight the significant effects*.

### fMRI

**Figure 5:**
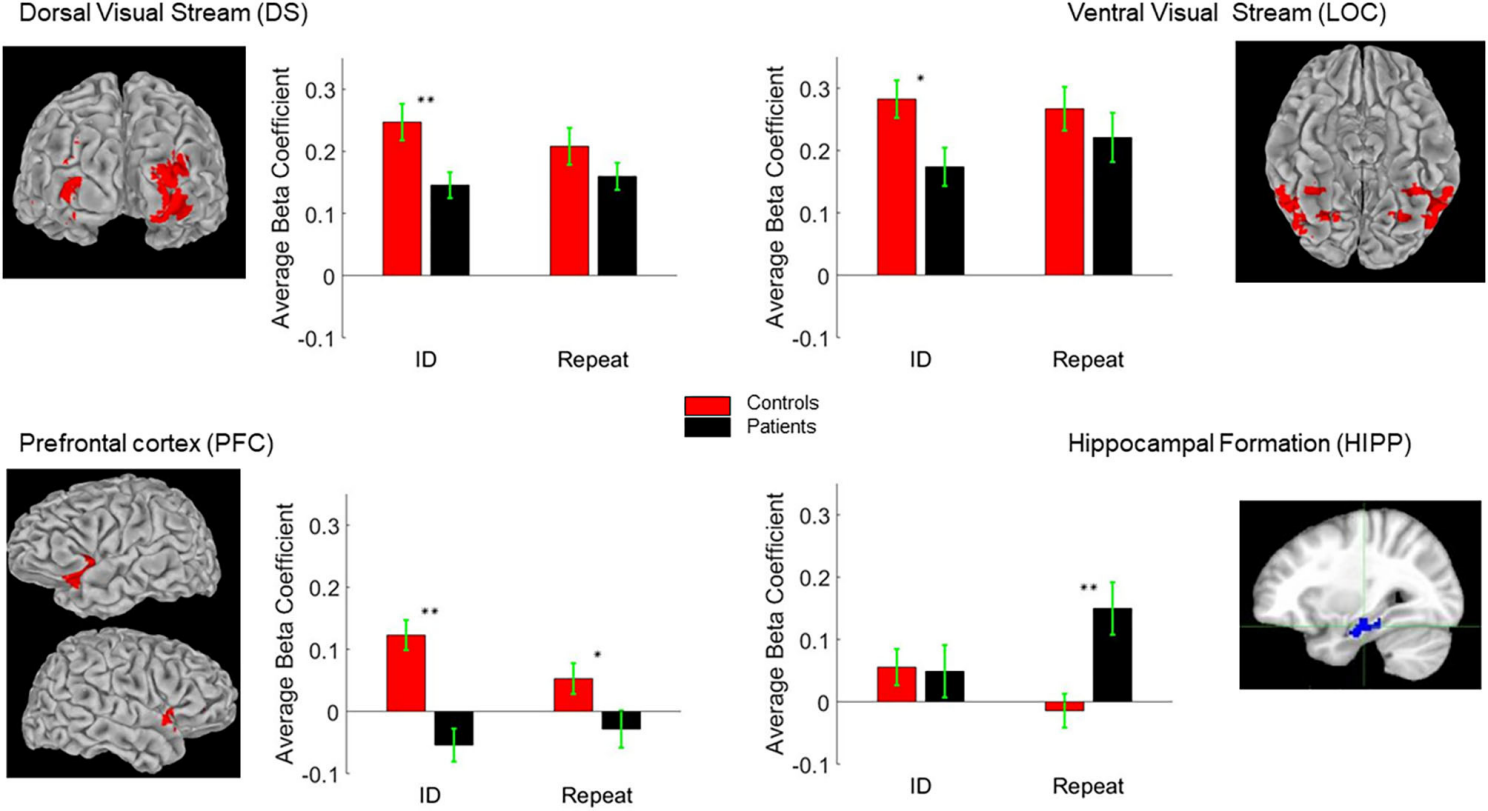
Regions of Interest (ROI) Talairach positions DS (±26, −79, 23), LOC (±27, −59, −8), PFC (±33, 4, 40) and HIPP (±26, −20, −20). The bar-charts show between group differences in BOLD response to ID and “Repeat” images (see text for details). *p < 0.05; **p < 0.01.

As in prior studies (6, 12), critical regions of activation for this task were in dorsal and ventral visual regions, as well as PFC and HIPP. We used the first level of analysis to delineate these ROIs (Figure 5). Activation patterns were therefore compared across these regions using the second level of analysis. As in previous studies using ERP and fMRI, no significant hemispheric differences were observed. We therefore collapsed the corresponding ROIs from each hemisphere for this analysis.

The second level of analysis in controls indicated significant activations in response to the novel stimuli in three of the four regions of interest namely the DS/BA19, PFC/BA47 and LOC/BA37 but not the HIPP/BA36. Significant activations in response to the primed stimuli in this group were also only observed in DS/BA19, PFC/BA47 and LOC/BA37. In the patients, however, the novel and the primed images resulted in significant activation of DS/BA19, LOC/BA37 and the HIPP/BA36 but not the PFC/BA47. Significant group differences were observed in DS/BA19, PFC/BA47 and LOC/BA37 for novel images. For primed images, the significant differences were primarily observed in PFC/BA47 and HIPP/BA36 (Table 2 and Figure 5).

**Table 2:** Significant fMRI closure activations in response to Novel and Primed images.

	**DS/BA19**	**PFC/BA47**	**LOC/BA37**	**HIPP/BA36**
**Patients**	***t*****, df**, ***p***	***t*****, df**, ***p***	***t*****, df**, ***p***	***t*****, df**, ***p***
ID (Novel Stimuli)	7, 18, <0.001	NS	5.7, 18, <0.001	1.2, 18, 0.26
Repeat (Primed) Stimuli	7.3, 18, <0.001	NS	5.6, 18, <0.001	3.6, 18, 0.002
**Controls**
ID (Novel Stimuli)	8.4, 20, <0.001	5.1, 20, <0.001	9.4, 20, <0.001	NS
Repeat (Primed) Stimuli	7, 20, <0.001	2.1, 20, 0.04	7.6, 20, <0,001	NS
**Group Differences**
ID (Novel Stimuli)	−2.8, 38, 0.009	−5.0, 38, 0.001	−2.5, 38, 0.02	NS
Repeat (Primed)	NS	−2.1, 38, 0.04	NS	3.3, 38, 0.003

**Figure 6:**
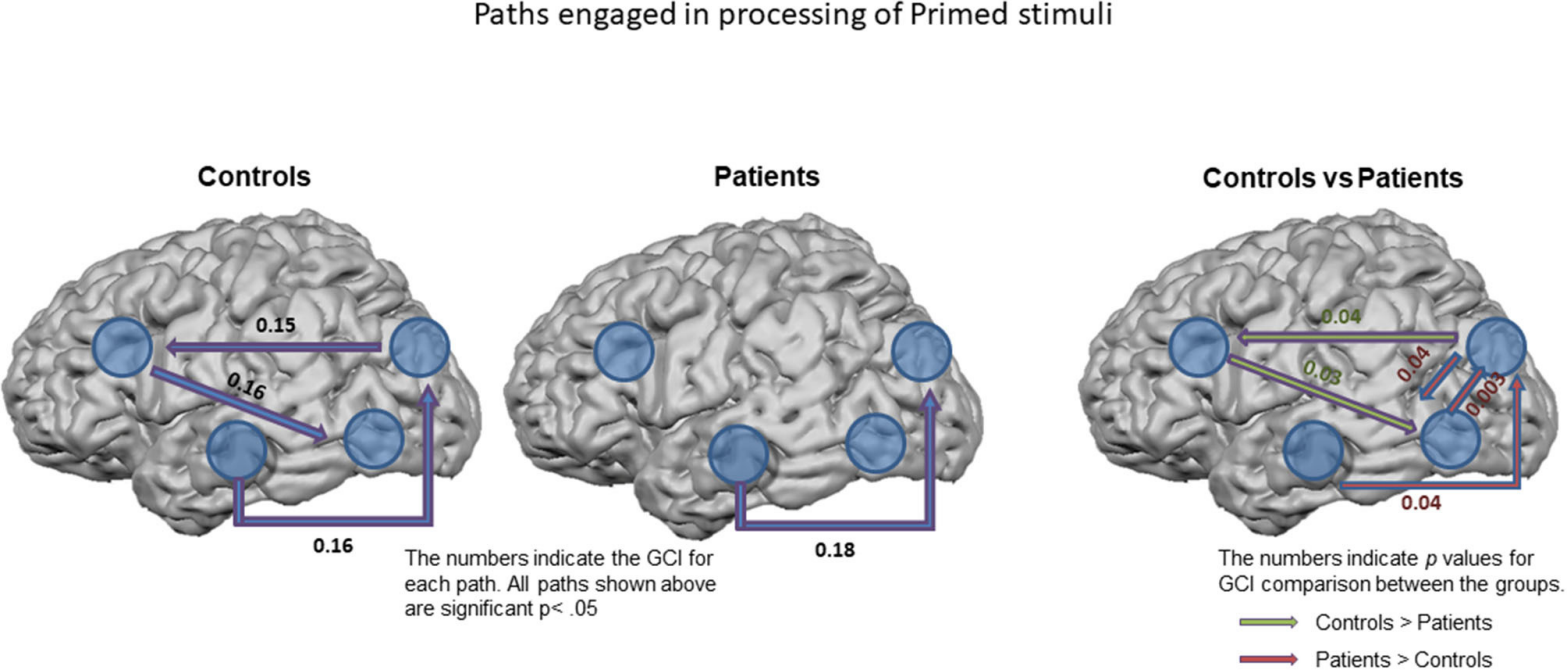
Paths engaged in processing of Primed stimuli. Granger Causality Index (GCI) used as a measure of functional connectivity across the ROIs in controls (left) and in patients (middle). All the paths shown are significant at *p* < 0.05. The right figure shows the significant differences observed between the groups. The numbers on the right figure represent significant *p*-values.

Directed functional connectivity measures in controls and patients indicated the path of activation of these regions significantly differed across the groups only for the primed images with greater inflow of information into the PFC from the DS and in turn from PFC to LOC in controls. Increased bidirectional flow of information between the DS and LOC as well as from HIPP to DS were observed in the patients (Figure 6) indicating that the patients utilize an alternate route of processing that is less reliant on PFC, where also the greatest group differences in closure processing were observed. The increased HIPP/DS and LOC/DS connectivity during closure of primed images suggests leveraging this circuitry in patients normalizes closure.

### Correlation Between ERP/fMRI/Clinical Measures

Patients showed significant reductions in POI (95.4 ± 17.4; *p* = 0.041), PSI (83.2 ± 8.7, *p* < 0.001), WMI (89.4 ± 10.0, *p* < 0.001) and BVMT-R (18.0 ± 8.2, *p* = 0.002) scores relative to published norms. Significant correlations were observed between the dorsal stream P1 amplitude for unprimed images with PSI and WMI. The P1 amplitude for primed images correlated significantly with POI and BVMT-R. No significant correlations between ventral stream Ncl or N1 amplitudes with neuropsychological measures were observed.

Similarly, reduced fMRI activation of the dorsal stream during closure of unprimed images correlated significantly with WMI (Table 3). Deficits in fMRI activation of the PFC during closure of unprimed images also correlated significantly with WMI and BVMT-R (Table 4). In contrast, no significant correlations were found during closure of primed images. Likewise, no significant correlations were observed between neuropsychological measures and ventral stream or HIPP activations, for closure of primed or unprimed images (all *p* > 0.15).

**Table 3:** Correlations between neuropsychological and electrophysiological measures in patients.

	**Unprimed Ncl**		**Primed Ncl**		**Unprimed P1**		**Primed P1**	
	***r***	***P*-value**	***r***	***P*-value**	***r***	***P*-value**	***r***	***P*-value**
Perceptual Organization Index	0.28	0.27	0.11	0.67	−0.17	0.52	0.51^*^	**0.037**
Processing Speed Index	−0.09	0.73	0.66	0.11	0.53^*^	**0.022**	0.019	0.94
Working Memory Index	−0.04	0.87	−0.12	0.65	0.52^*^	**0.034**	−0.68	0.003
Brief Visual Memory Test	0.004	0.99	−0.063	0.8	−3.1	0.22	–0.6^*^	**0.008**

* *p < 0.05*.

*Bold values highlight the significant effects*.

**Table 4:** Correlations between neuropsychological and fMRI measures in patients.

	**PFC**				**Dorsal Stream**			
	**Unprime Closure**		**Prime Closure**		**Unprime Closure**		**Prime Closure**	
	***r***	***P*-value**	***r***	***P*-value**	***r***	***P*-value**	***r***	***P*-value**
Perceptual Organization Index	0.37	0.16	−0.31	0.24	−0.29	0.28	-0.10	0.72
Processing Speed Index	0.05	0.85	−0.29	0.28	−0.29	0.28	−0.21	0.44
Working Memory Index	0.546^*^	**0.03**	0.02	0.95	0.64^**^	**0.01**	−0.32	0.23
Brief Visual Memory Test	0.610^*^	**0.01**	0.04	0.89	−0.42	0.11	−0.46	0.08

* *p < 0.05*;

** *p < 0.01*.

*Bold values highlight the significant effects*.

## Discussion

We have previously demonstrated deficits in perceptual closure processes in schizophrenia using behavioral (4, 5), ERP (4, 6) and fMRI (6) measures. These studies suggested that dysfunction in early dorsal visual pathway significantly contribute to the failure of more complex perceptual processes (39). Nonetheless, previous behavioral studies (5, 40–42) have shown that patients derive benefit comparable to control participants from prior exposure to the fragmented images, although underlying mechanisms of this preserved effect were not determined. This study builds on these prior studies and extends them by first, combining ERP findings with results of parallel fMRI investigation to study the mechanisms of intact perceptual priming in patients, while second, providing a direct between-group comparison of fMRI functional connectivity patterns in patients and controls. Finally, neuropsychological data were collected to enable the characterization of the functional neuroanatomy of perceptual priming processes more fully within the context of neuropsychological dysfunction in schizophrenia.

As in a previous study (21) repetition effects were manifest as a larger N1 to repeated vs. novel stimuli in controls. Here we show this differential effect is intact in patients and the magnitude of the N1 is significantly correlated with the magnitude of the Ncl pointing to recursive interactions between sensory and perceptual level processes (11). We have previously demonstrated that closure-related recursive processes involve interactions between dorsal visual stream, PFC, HIPP and ventral visual stream (12, 13). As previously, these regions showed significant activations for closure of novel images with significant group differences at dorsal and ventral streams and PFC but not HIPP. In the processing of primed images however, significant differences between groups were observed at PFC and HIPP with patients showing significantly greater activation of HIPP and no significant activation of PFC, a pattern that was exactly reversed in controls. Significant differences in functional connectivity across the groups were observed for the primed images which also indicated that patients utilize an alternate route of processing that is less reliant on PFC. We (13) as well as others (43, 44) have previously suggested that the magnocellular system provides rapid low-resolution input to the frontal cortex, which then helps trigger top-down object recognition. Within such a framework, the lower reliance on PFC could be a consequence of avoiding a less effective dorsal stream-PFC information processing stream as well as intrinsic abnormalities in the prefrontal function in schizophrenia.

Finally, this study assesses closure-related priming activity relative to traditional neuropsychological measures. In the present sample, consistent with prior publications (45), significant reductions in PSI were observed relative to normative values (*p* < 0.001). PSI along with POI are two components that make up the WAIS performance IQ. Unlike in a previous study (6), in the present population of patients, POI was within the normal range which could explain the lack of correlations between abnormalities in POI and Ncl/N1 indices of closure/priming. Nevertheless, for unprimed images, impaired P1 generation correlated significantly with performance on both PSI and WMI. Similarly, PFC activation to unprimed stimuli correlated significantly to WMI, suggesting that dorsal stream inputs to PFC may be important to mnemonic function. By contrast, P1 to primed stimuli correlated significantly to POI and BVMT-R, suggesting that the P1 modulation in response to priming may contribute significantly to higher order visual functions.

Here we used GCM to explore directed influences to and from our ROIs. This approach uses the temporal information in two stochastic time-series and, by determining temporal precedence, infers the directionality of the functional connections. It is therefore not reliant on a priori models used in approaches such as dynamic causal modeling (DCM) (46). It is important to acknowledge the latency differences in HRF across different brain regions (47), the low-pass filtering and the temporal down-sampling inherent in the hemodynamic response observed in fMRI. Having said that, multiple previous studies (25, 35, 47, 48) have shown the viability of Granger causality in measuring directed functional connectivity in fMRI. Moreover, in this study, and in our previous investigations of closure process (5, 6, 12, 13, 21) we have provided converging evidence that suggests the effects observed here are physiological in origin and not an epiphenomenon of the imaging method. Here we also used the dGCM approach that to the extent possible, address some of these issues (25, 48). Nevertheless, the GCM approach alone would not be sufficient to determine effective connectivity (49, 50). It does however serve two important functions: First being its ability to distinguish between normal and abnormal patterns of large-scale cortical network interactions (49, 51), similar to other functional connectivity measures but with the added information of directionality. This function is thus very helpful in characterizing the network-level dysfunctions in neuropsychiatry. Second, it can provide valuable information for physiologically informed dynamic causal modeling and aid in the hypothesis driven effective connectivity investigations (52). In this study, we believe the use of a multimodal approach (EEG and fMRI) provides further convergent information in the context of the two above mentioned points.

The study is potentially limited by the small sample size, which suggests that replication in a larger group of subjects may be warranted, and by the relatively high doses of antipsychotic medication. However, no correlation was observed between any of the dependent measures and chlorpromazine equivalents, which argues against direct medication effects. Additionally, a more balanced sex distribution would have been preferable. However, previous studies of closure with a more balanced population (5, 12) indicated no sex differences in the performance of this task.

In summary, this study, to our knowledge, presents the first multi-modal investigation of priming processes in perceptual closure and underscores the importance of network dynamics in pathophysiology of cognitive processes in schizophrenia. We demonstrate that whereas closure processes that require information transfer from dorsal visual pathway to PFC are impaired, those that rely solely on processing within LOC and its interaction with HIPP are intact and underlie the preserved priming effect in schizophrenia. The increased connectivity in hippocampal/dorsal-visual and ventral/dorsal-visual pathway could be further investigated using neuromodulatory approaches to study the possibility of improving closure processing in patients. Overall, these findings reinforce the importance of early dorsal stream visual dysfunction to impaired cognitive processing and suggest that impaired rapid input of visual information *via* the dorsal stream to cognitive brain regions such as PFC and HIPP may contribute significantly to the overall pattern of cognitive dysfunction in schizophrenia.

## Significance Statement

Deficits in both auditory and visual sensory processing are a core feature of schizophrenia and contribute significantly to impaired functional outcome. Within the visual system, deficits are most apparent within the subcortical magnocellular visual pathway, which projects low resolution object information rapidly to prefrontal cortical areas *via* the dorsal visual stream. We have previously shown that when processing fragmented pictures, schizophrenia patients require more information to successfully “close” the images, but paradoxically show intact ability to benefit from stimulus repetition. Here we investigated underlying neural substrates using a multimodal ERP and fMRI approach. Consistent with a priori predictions, patients showed significant impairments in dorsal stream activation to novel stimuli as reflected in both ERP and fMRI, and absence of the normal correlation between dorsal stream and prefrontal activity. By contrast, modulation of activity in the ventral visual stream lateral occipital complex by stimulus repetition was intact. In functional connectivity analyses, controls showed significant operation of the dorsal stream-PFC-ventral stream pathway, whereas patients did not. Overall, these findings support a model in which loss of rapid, low resolution information to prefrontal cortex *via* the dorsal stream undermines novel object recognition in schizophrenia and illustrate how dysfunction within early sensory pathways, such as the visual magnocellular/dorsal stream pathway, contributes to higher order cognitive dysfunction.

## Data Availability Statement

The raw data supporting the conclusions of this article will be made available by the authors, without undue reservation.

## Ethics Statement

The studies involving human participants were reviewed and approved by Institutional Review Board, Nathan Kline Institute for Psychiatric Research. The patients/participants provided their written informed consent to participate in this study.

## Author Contributions

PS and DJ contributed to design, implementation, analysis, and manuscript preparation. AB, DA, ZW, HD, GS, and AM contributed to data analysis and implementation. All authors contributed to the article and approved the submitted version.

## Conflict of Interest

DJ reports Intellectual property for NMDAR agonists in schizophrenia, NMDAR antagonist in depression, fMRI for prediction of ECT response, and ERP biomarkers for diagnosis of Alzheimer disease and schizophrenia. Equity in Glytech, AASI, and NeuroRx. Scientific advisory board NeuroRx, Promentis. Consultant payments Concert, Lundbeck, Phytec, Autifony, SK Life Sciences, Biogen, Cadence, and Pfizer. The remaining authors declare that the research was conducted in the absence of any commercial or financial relationships that could be construed as a potential conflict of interest.
